# Evaluating Compliance With the Ottawa Rules: A Retrospective Clinical Audit at a District General Hospital in the UK

**DOI:** 10.7759/cureus.65115

**Published:** 2024-07-22

**Authors:** Saif Dalla Ali, Omar A Alhiraki, Tahir Naeem

**Affiliations:** 1 General Surgery Department, Lincoln County Hospital, Lincoln, GBR; 2 Acute Medicine Department, Lincoln County Hospital, Lincoln, GBR; 3 Radiology Department, Lincoln County Hospital, Lincoln, GBR

**Keywords:** ottawa, ankle, knee, clinical audit, emergency department, x-ray, compliance

## Abstract

Background: The Ottawa Rules are clinical decision tools designed to assist healthcare providers in determining the need for radiographs in patients with ankle or knee injuries. Compliance with these rules can lead to more efficient use of resources and reduced radiation exposure.

Objective: This retrospective clinical audit aimed to evaluate healthcare provider's compliance with the Ottawa Rules in an Emergency Department setting and assess the positivity rates of requested knee and ankle X-rays.

Methods: A two-cycle retrospective audit was conducted at Lincoln County Hospital's Emergency Department, involving 648 X-rays collected in two cycles. In between, multiple interventions were implemented to improve the outcomes.

Results: The study revealed varying levels of compliance with the Ottawa Rules, with higher compliance observed for knee X-rays than ankle X-rays. The compliance for knee X-rays improved from 74.6% to 89.9% and ankle X-rays improved from 33.1% to 75.8%. Positivity rates for ankle radiographs were higher than knee radiographs in both cycles. The interventions implemented between the cycles significantly improved compliance rates with the Ottawa Rules.

Conclusion: The findings underscore the importance of adherence to the Ottawa Rules in optimizing patient care and resource utilization. The study suggests the need for continued education and periodic audits to maintain and further improve compliance rates. Additionally, the higher positivity rates for ankle radiographs highlight the importance of targeted imaging strategies based on clinical guidelines.

## Introduction

Managing a significant patient load necessitates the implementation of clear and concise guidelines to facilitate healthcare providers' decision-making. These guidelines are instrumental in improving the quality and efficiency of patient care while optimizing cost-effectiveness [1]. Ankle and knee injuries are among the most common musculoskeletal injuries, affecting people of all ages and activity levels [2,3]. When patients present with ankle or knee injuries, the need for proper tools to help the decision to order an X-ray or not is crucial in providing appropriate care and avoiding unneeded radiation exposure. The Ottawa Ankle Rules is a clinical decision tool that has been developed to assist clinicians in deciding whether an X-ray is needed. Compliance with these rules ensures effective and efficient diagnosis while minimizing patient discomfort, healthcare costs, and radiation exposure [4,5].

According to Ottawa Rules, for ankle injuries, X-rays are recommended only if there is pain in the malleolar zone accompanied by tenderness at the posterior edge or tip of the lateral or medial malleolus or if the patient is unable to bear weight immediately in the Emergency Department (ED) [6,7]. For knee injuries, X-rays are indicated if the patient is aged 55 years or older regardless of other factors, has tenderness at the head of the fibula, isolated tenderness of the patella, inability to flex the knee to 90 degrees, or an inability to bear weight (taking four steps) immediately and upon presentation [8,9].

Research has demonstrated the Ottawa Rules as a highly effective clinical tool for ruling out fractures, boasting near-perfect sensitivity and average specificity [4,10-12]. Their implementation can reduce unnecessary radiographs by 30-40% [4]. The National Institute for Health and Care Excellence (NICE) guidelines incorporate these rules as essential tools for assessing the need for radiographs in patients with suspected fractures [4]. Therefore, these tools have been widely used by healthcare providers in the UK; subsequently, the Royal College of Radiologists (RCR) has promoted auditing their use [13].

This paper presents a closed-loop audit conducted to assess healthcare providers' compliance with the Ottawa Rules in the ED at Lincoln County Hospital in case of requesting X-rays for suspected knee and ankle injuries and the positivity rates of requested scans. This audit's importance lies in ensuring the efficient utilization of the available validated tools, contributing significantly to the growing body of quality improvement initiatives in the National Health Service (NHS) and, eventually, its potential to enhance patient care quality.

## Materials and methods

Study design and sampling

This is a closed-loop audit, consisting of two cycles. Both cycles were cross-sectional, retrospective studies, that were conducted at the ED of Lincoln County Hospital, a District General Hospital (DGH) in East Midlands, UK. A total of 648 X-rays were reviewed. The first cycle encompassed 388 radiographs collected from April 2023 to July 2023, while the second cycle included 260 X-rays collected from December 2023 to February 2024. The ED at Lincoln County Hospital is a hub for many patients referred from nearly every speciality in the area. It offers various diagnostic imaging services and daily assessments of many patients with knee and ankle injuries.

Inclusion and exclusion criteria

The inclusion criteria for the study were patients aged 18 years or older who underwent knee or ankle X-rays in the ED or Urgent Treatment Centre (UTC) during the selected periods. We excluded patients whose medical records lacked complete documentation, those who underwent post-fracture reduction manoeuvres, patients under the age of 18, and patients who could not be examined adequately due to intoxication, multiple painful injuries, or unconsciousness.

Ethical considerations

Ethical approval was obtained from the ULHT Clinical Governance Department. The first audit cycle was registered under the project (No. L0928), and the second was registered under the project (No. L1116).

Data extraction

Raw data were obtained using the Hospital's Picture Archiving and Communication System (PACS), which was used to retrieve and review X-rays, request information, reports, and patient records. The data were then cleared according to inclusion and exclusion criteria, coded in Microsoft Excel, and analyzed later via Statistical Package for the Social Sciences (IBM SPSS Statistics for Windows, IBM Corp., Version 26.0, Armonk, NY). Data were assessed across seven domains: (1) age; (2) gender; (3) type of X-ray; (4) clinical History; (5) compliance with Ottawa Rules; (6) radiologist report; (7) positive results. Compliance was defined as adherence to one of the points of relevant rules, and positivity was defined as any significant positive finding mentioned in the X-ray results report.

Statistical analysis

Data analysis was performed using SPSS. Descriptive and inferential statistical tests were used to analyze the data and associations between variables. Descriptive statistics evaluated the demographic characteristics, adherence, and positivity rate. Inferential statistics, including the chi-square test, assessed the factors associated with adherence to the Ottawa Rules and the positivity rate, with p < 0.05 considered the statistically significant cut-off.

Interventions

After the first cycle (August 2023), we implemented several interventions to enhance compliance with the Ottawa Rules in our hospital. The interventions were an educational session to familiarize healthcare providers with the significance of adherence to the Ottawa Rules, the distribution of educational leaflets, informative posters placed in the ED and UTC as constant reminders of the rules, and enlightening emails sent to healthcare providers to strengthen compliance with the Ottawa Rules.

Outcomes

The primary aim of this audit was to assess adherence to the Ottawa Rules among healthcare providers. We hypothesized that adherence was insufficient and aimed to enhance it through several proposed interventions and to re-audit via a second cycle. The secondary objective was to ascertain the positivity rate of the requested X-rays.

## Results

First cycle

A total of 388 X-rays were included in our study. Of these, 247 (63.7%) were knee X-rays and 141 (36.3%) were ankle X-rays. Upon further analysis of the data, the mean age of the patients in the study was 60.98 (standard deviation (SD)=21.8), of whom 219 (56.4%) were females. The adherence to the Ottawa Rules in general was 59.5%, and 23.3% of the tests yielded positive results, which is further summarised in Table 1.

**Table 1:** Patients’ characteristics, compliance with Ottawa Rules, and respective radiographic interpretations (first cycle) * means having a valid clinical justification for requesting an X-ray that is not based on the Ottawa Rules.

Characteristics		Frequency	Percentage
Gender	Male	169	43.6
	Female	219	56.4
Age	<55 yo	140	36.6
	≥55 yo	243	63.4
	Missing	5	-
X-ray Type	Right Knee	127	32.7
	Left Knee	120	30.9
	Right Ankle	72	18.6
	Left Ankle	69	17.8
Image Category	X-ray Knee	247	63.7
	X-ray Ankle	141	36.3
Compliance With Ottawa Rules	Yes	256	59.5
	No	127	33.2
	Reasonable*	28	7.3
	Missing	5	-
Report (Image Results)	Positive	80	23.2
	Negative	265	76.8
	Missing	43	-

The compliance percentage in knee X-rays was 74.6% (n=182) compared to ankle X-rays, which yielded a lower compliance rate of only 33.1% (n=46), which is statistically significant (χ²=63.5, p<0.0001). Age delineation showed that 22.5% (n=31) of X-ray orders for patients aged below 55 years were compliant with the Ottawa Rules, compared to 80.1% (n=193) in those aged 55 and above, showing a significant association (χ²=120.8, p<0.0001). Gender-based analysis revealed 57% (n=69) compliance in males' X-rays and 60.8% (n=132) in females, with no statistically significant difference (Table 2).

**Table 2:** Compliance with Ottawa Rules and positivity rate of X-ray findings by X-ray type, age, and gender (first cycle) * indicates statistically significant.

	Frequency (%)	Pearson's Chi-square	P-value
Compliance with Ottawa Rules			
X-ray Knee	182 (74.6%)	63.5	<0.0001*
X-ray Ankle	46 (33.1%)		
<55 Y	31 (22.5%)	120.8	<0.0001*
≥55 Y	193 (80.1%)		
Male	69 (57%)	0.37	0.83
Female	132 (60.8%)		
Reporting positivity			
X-ray Knee	39 (18.0%)	8.93	0.004*
X-ray Ankle	41 (32.0%)		
<55 Y	25 (207%)	0.66	0.5
≥55 Y	55 (24.6%)		
Male	23 (15.8%)	7.85	0.006*
Female	57 (28.6%)		

We found that 18% (n=39) of knee X-rays were positive, compared to 32% (n=41) of ankle X-rays, which is a statistically significant result (χ²=8.93, p=0.004). Conversely, ankle X-rays revealed a positivity rate of 32.0%. Stratifying by age, 20.7% (n=25) of patients below 55 years displayed positive findings, while this rate was 24.6% (n=55) for those aged 55 and above, with the younger cohort showing no significant difference (χ²=0.66, p=0.5). A gender disparity was observed: males showed a positivity of 15.8% while females displayed a rate of 28.6% (χ²=7.85, p=0.006), all illustrated in (Table 2).

Our findings in the first cycle revealed a statistically significant association between adherence to the Ottawa Rules and positive outcomes (x=9.7, p=0.007). Specifically, there was a 22.6% positivity rate when following the Ottawa Rules, in contrast to an 18.9% rate when not adhering (Table 3).

**Table 3:** Compliance with Ottawa Rules to reports (images’ results) cross-tabulation (first cycle)

			Report (Image Results)		Total
			Positive	Negative	
Compliance with Ottawa Rules	Yes	Count	47	161	208
		% within compliance with Ottawa Rules	22.6%	77.4%	100%
	No	Count	21	90	111
		% within compliance with Ottawa Rules	18.9%	81.1%	100%
	Reasonable	Count	12	13	25
		% within compliance with Ottawa Rules	48.0%	52.0%	100%

Second cycle

In the second cycle, we included 260 X-rays, of which 169 (65%) were knee X-rays and 91 (35%) were ankle X-rays. Age distribution showed that most patients were aged 55 and above, accounting for 65.9% of the patients. Remarkably, compliance with the Ottawa Rules was high, with 85% (221) of the X-rays meeting the rules. Regarding X-ray positivity, 27.7% of the X-rays showed positive findings (72 positive cases), while 188 cases (72.3%) were negative (Table 4).

**Table 4:** Patients’ characteristics, compliance with Ottawa Rules, and respective radiographic interpretations (second cycle)

Characteristics		Frequency	Percentage
Gender	Male	111	42.7
	Female	149	57.3
Age Categories	Less than 55	87	34.1
	55 and above	168	65.9
	Missing	5	-
Images Category	Knee X-ray	169	65.0
	Ankle X-ray	91	35.0
Meet the Ottawa Rules	Yes	221	85.0
	No	39	15.0
Report (Image Results)	Positive	72	27.7
	Negative	188	72.3

When further analyzing the data, it has been noted that compliance with the Ottawa Rules varied across various categories of X-rays, age, and gender. The compliance rate with the Ottawa Rules was 89.9% for knee X-rays and 75.8% for ankle X-rays. This difference was statistically significant (χ²=9.24, p=0.003). Age played a crucial role in clinicians' compliance with the knee rules, which showed a rate of 69.9% for patients under 55, significantly different from those aged 55 and above (χ²=27.1, p=0.001). Gender-based analysis revealed no significant statistical difference (χ²=0.681, p=0.48), as 82.9% of males and 86.6% of females' X-rays were compatible with the Ottawa Rules (Table 5).

**Table 5:** Compliance with Ottawa Rules and positivity rate by X-ray type, age, and gender (second cycle) * indicates statistically significant

	Frequency (%)	Chi-square	P-value
Compliance with Ottawa Rules			
X-ray Knee	152 (89.9%)	9.24	0.003*
X-ray Ankle	69 (75.8%)		
<55 Y	60 (69.9%)	27.1	0.001*
≥55 Y	157 (93.5%)		
Male	92 (82.9%)	0.681	0.48
Female	129 (86.6%)		
Reporting Positivity			
X-ray Knee	32 (18.9%)	18.4	0.001*
X-ray Ankle	40 (44%)		
<55 Y	19 (21.8%)	2.08	0.09
≥55 Y	51 (30.4%)		
Male	35 (31.5%)	1.4	0.26
Female	37 (24.8%)		

The positivity rates of X-ray findings across different categories demonstrated that for knee X-rays, the positivity rate was 18.9% compared to ankle X-rays (44.0%), which is statistically significant (χ²=18.4 and p=0.001). When analyzed by age groups, the positivity rate was 21.8% for patients under 55 and 30.4% for those 55 and older, with no significant association identified (χ²=2.08, p=0.09). Regarding gender, males showed a positivity rate of 31.5%, while females had a rate of 24.8%, with no significant association observed (χ²=1.4, p=0.26) (Table 5).

**Figure 1:**
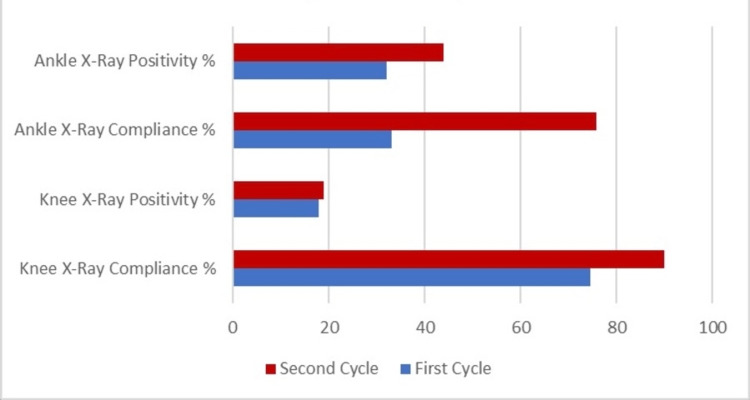
Compliance with Ottawa Rules and positivity rate by X-ray type

After implementing the interventions and completing the second cycle, a comparative analysis of the results of both cycles was conducted. In the initial cycle, the compliance rate for knee X-rays was 74.6%, which significantly improved in the second cycle to 89.9%. Ankle X-ray compliance improved from 33.1% in the first cycle to 75.8% in the second. This marked improvement indicates successful interventions, likely attributable to the increased emphasis on training and awareness of the Ottawa Rules among the medical staff. The second cycle showed no statistically significant association between adherence and positivity. The positivity rate for findings in knee X-rays slightly increased from 18.0% in the first cycle to 18.9% in the second. In contrast, ankle X-rays showed a significant increase in positivity from 32.0% to 44.0% (Figure 1). This suggests that while the overall compliance improved, the specificity of knee X-rays could still be optimized to avoid unnecessary imaging.

## Discussion

The Ottawa Knee Rule was developed in 1995 and has been extensively tested and shown to improve patient care quality, reduce unnecessary radiographs, and increase cost-effectiveness [14,15]. Similarly, the Ottawa Ankle Rules were developed earlier in 1992. Multiple studies have evaluated its benefits and proven its reliability and accuracy [4,16]. Those rules are a part of the NICE guidelines’ recommendation [17]. Appropriate application of the rules can reduce the number of radiographs performed in the setting of acute knee and ankle injuries, with the potential benefits of improved cost efficiency and decreased waiting time for the patients [14]. There is a lack of research on the local compliance with these rules in the UK, specifically at United Lincolnshire Hospitals NHS Trust. Thus, we conducted a two-cycle audit to increase compliance and raise awareness about the Ottawa Rules within our institution. The results of our study have concluded that the compliance rate has improved in the second cycle, which points out that the actions we have implemented were successful. Such strategies have been documented in the literature to enhance guideline adherence and optimize clinical practice effectively [18,19].

Diving deeper into the results, both cycles showed higher compliance with the knee X-ray rule than the ankle X-ray rule. This difference can be attributed to including the patient’s age as a criterion in the Ottawa Knee Rule, unlike the Ottawa Ankle Rule. Age was found to significantly influence compliance rates, with the first cycle showing 80.1% compliance in patients aged 55 and above and 22.1% in those below 55. This trend persisted in the second cycle, where most patients were 55 and above. These findings suggest that further research is needed to address this issue, particularly considering the age factor. Literature has highlighted age as a factor affecting the sensitivity and specificity of the Ottawa Rules. Some research advises cautious use of the Ottawa Knee Rules in patients under 18 years old [20]. In another study on the utility of the Ottawa Ankle Rules in an ageing population, they found evidence supporting the addition of an age criterion to enhance rule effectiveness [21].

Previous studies have demonstrated that the Ottawa Ankle and Knee Rules have high sensitivity and low to medium specificity [12,14]. In the first cycle, our findings indicated a statistically significant association between adherence to the Ottawa Rules and positive outcomes. Specifically, the positivity rate was 22.6% when the Ottawa Rules were followed, compared to 18.9% when adherence was low. Further analysis showed the positivity of ankle radiographs was 32% compared to knee radiographs (18%) with similarities in the second cycle. Although compliance was higher when ordering knee X-rays, we observed that the positivity rate was higher in patients who underwent ankle X-rays following a decision aided by the Ottawa Rules. Those findings can be attributed to many factors, including the varying specificity levels of these clinical decision rules utilized for each joint [22,23].

Further research into the specific types of injuries captured by each set of rules could provide additional insights into the difference in positivity rates. The patient's age was another influencing factor for the discrepancy in positivity, as there were more knee X-ray requests, potentially leading to more unnecessary knee X-rays. This suggests a possible age bias in the decision-making process for knee X-rays. The Ottawa Knee Rule has been compared to the Pittsburgh Decision Rule, another validated rule. A comparison of the two rules revealed that both had high sensitivities, although the Pittsburgh Decision Rule was significantly more specific [24]. The validity of the Ottawa Ankle Rules after more than 20 years of use has sparked a debate on whether to continue or replace them [25].

Limitations

Cross-sectional and retrospective study designs limit our ability to demonstrate causality and are prone to selection biases, which may affect the generalizability and accuracy of our findings. Compliance was determined by radiology requests only as data collection was solely from the online system (PACS), without reviewing patient notes, potentially introducing surveillance bias. This audit did not evaluate the Foot Ottawa Rules; we only assessed the ankle.

## Conclusions

Our audit highlights the importance of adhering to the Ottawa Rules in the ED, demonstrating possible benefits for patient care and efficient resource utilization. The interventions implemented between the first and second cycles significantly improved compliance with the Ottawa Rules. Additionally, the increase in positivity rates for ankle X-rays indicates a more targeted and justified use of imaging. Continued education and periodic audits are recommended to maintain and build on these improvements.
